# Complete genome sequence of a novel secovirid infecting cassava in the Americas

**DOI:** 10.1007/s00705-021-05325-2

**Published:** 2022-01-03

**Authors:** Ana M. Leiva, Jenyfer Jimenez, Hector Sandoval, Shirley Perez, Wilmer J. Cuellar

**Affiliations:** 1 Virology Laboratory, Cassava Program, Crops for Nutrition and Health, International Center for Tropical Agriculture, Palmira, Colombia; 2 AGROSAVIA, Centro de Investigacion La Libertad, Sede Yopal, Colombia

## Abstract

We report the complete genome sequence of a field isolate of a novel bipartite secovirid infecting cassava in Colombia, provisionally named "cassava torrado-like virus" (CsTLV). The genome sequence was obtained using Oxford Nanopore Technology, and the 5’ ends were confirmed by RACE. The RNA1 is 7252 nucleotides (nt) long, encoding a polyprotein of 2336 amino acids (aa) containing the typical “replication block”, conserved torradovirus motifs, and a Maf/Ham1 domain, which is not commonly found in viral genomes. The RNA2 is 4469 nt long and contains two overlapping ORFs encoding proteins of 226 and 1179 aa, showing the characteristic genome arrangement of members of the genus *Torradovirus*.

**Supplementary Information:**

The online version contains supplementary material available at 10.1007/s00705-021-05325-2.

Cassava torrado-like virus (CsTLV) is a partially characterized member of the family *Secoviridae* [1, 2] that was originally detected in cassava plants (*Manihot esculenta* Crantz) displaying root symptoms of cassava frogskin disease (CFSD) [3], an endemic disease of cassava in the Americas that can severely affect the root yield of the crop [4]. The virus is found in mixed infections [3, 5] and can induce leaf chlorotic spots symptoms in single infections in cassava [2]. Maf/HAM1 proteins are nucleoside triphosphate (NTP) pyrophosphatases that reduce mutagenesis by intercepting non-canonical NTPs and preventing their incorporation into DNA or RNA. They are highly conserved in prokaryotes and eukaryotes [6], but there are only a couple of examples of their presence in viral genomes [7, 8].

The complete genome of CsTLV was obtained from a cassava plant of the commercial variety Melua-31, collected in August 2020 in Yopal, Colombia, and total RNA extraction with CTAB was done as reported previously [9]. For library preparation, we followed the SQK-DCS109 protocol (Oxford Nanopore Technologies), which uses RNase Cocktail Enzyme Mix (Thermo Fisher) to eliminate ribosomal RNA. The library was loaded onto a FLO-MIN106 R9.4 flowcell and sequenced for 48 h in a MinION using MinKnow software v2.0. Basecalling was performed using Guppy v5.0.11, and assembly was performed as described by Leiva *et al*. [10]. The quality of the consensus sequence obtained was checked using Qualimap v2.2.1 [11], and low-coverage regions and 5’ ends were confirmed by sequencing of overlapping RT-PCR products and using a 5' RACE System for Rapid Amplification of cDNA Ends (Invitrogen).

Excluding the poly-A tail, the RNA1 (OK040225) is 7252 nt long and has a single open reading frame encoding a polyprotein of 2336 aa (260 kDa). It contains the typical “replication block” and a Maf/Ham1 domain at its 3’ end at aa 2165 (Fig. 1A). The RNA2 (OK040226) is 4469 nt long and contains two ORFs: RNA2-ORF1 is 678 nt long and encodes a predicted protein of 226 aa (25 kDa). RNA2-ORF2 is 3534 nt long and overlaps with 44 nt of the 3’ end of RNA2-ORF1; it encodes a predicted polyprotein of 1179 aa (131 kDa) containing the 3A/RNA2 movement protein domain (A3) and three coat proteins domains. The sequence obtained showed more than 98% aa sequence identity to all other partial CsTLV sequences available in the GenBank database, and BLASTp analysis revealed the highest aa sequence identity to squash chlorotic leaf spot virus (NC_035221; NC_035222), with 37.75% aa sequence identity and 82% coverage for RNA1 and 45.53% sequence aa identity and 77% coverage for RNA2.

**Fig. 1:**
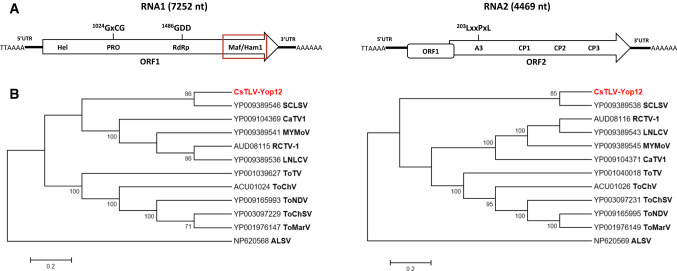
(A) Genome organization of CsTLV isolate Yop12 showing the location of conserved consensus motifs shared by torradoviruses [1] and the Maf/Ham1 domain (red frame). (B) Phylogenetic relationships of CsTLV to members of the genus *Torradovirus*, analyzed using the aa sequences of RNA1 and RNA2 polyproteins. The phylogenetic tree was generated by the neighbor-joining method using MEGA. The evolutionary distances were computed using the Poisson correction method (number of aa substitutions per site).

Maf/Ham1 domains have only been reported in potyvirids. The first one was discovered by Mbanzibwa *et al*. [7] while working on the characterization of a Tanzanian isolate of cassava brown streak virus (genus *Ipomovirus*) (GenBank accession no. FJ039520.1), and a second one was reported by Knierim *et al*. [8] in a German isolate of euphorbia ringspot virus (genus *Potyvirus*) (GenBank accession no. NC_031339.1) (Fig. 2). Recently, Tomlinson *et al*. [12] demonstrated the activity of the potyvirid Maf/Ham1 in yeast and uncovered their role as a necrosis determinant in *Nicotiana benthamiana*, and James *et al*. [13] have suggested possible functions for this domain. In conclusion, CsTLV is an atypical secovirid encoding a Maf/Ham1 protein domain, which has been described so far only in viruses infecting euphorbiaceous hosts [2, 7, 8]. Further studies are underway to determine the biological role of CsTLV Maf/Ham1 in the virus infection cycle in cassava.

**Fig. 2:**
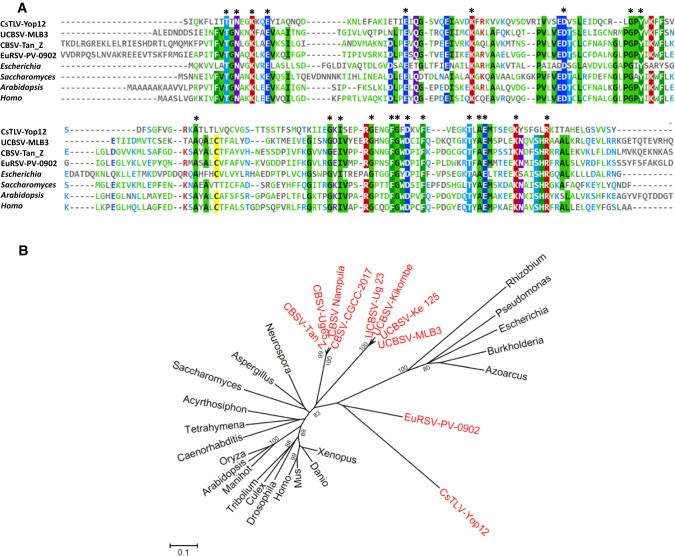
(A) Sequence alignment showing the conserved sites of Maf/Ham1 domains from different taxa. The figure was made using Mview [14]. (B) Phylogenetic relationships of the Maf/Ham1 domain. Viruses (in red): EuRSV-PV-0902, YP_009305422; CBSV-Nampula, AYW01246; CBSV-Tan_Z, ACT78701; CBSV-Ug65, QGW67508; CBSV-CGCC-2017, QCR98745; UCBSV-Kikombe, ARQ80023; UCBSV-MLB3, ACM48176; UCBSV-Ug_23, CBA18486; UCBSV-Ke_125, ASG92173; CsTLV-Yop12, **??**OK040225**??**; *Escherichia* 1K7K_A; *Pseudomonas*, WP_011064019; *Burkholderia*, KHS13049; *Azoarcus*, CAL96580; *Rhizobium*, CAK05869; *Saccharomyces*, CAA89597; *Aspergillus*, XP_754075; *Neurospora*, XP_955963; *Arabidopsis*, NP_567410; *Oryza*, XP_015613001; *Manihot*, XP_021594792; *Caenorhabditis*, AAL14111; *Tetrahymena*, XP_977249; *Acyrthosiphon*, NP_001233079; *Drosophila*, EDV32196; *Culex*, XP_038111262; *Tribolium*, XP_974197; *Xenopus*, AAI10772; *Danio*, NP_001093456; *Mus*, EDL28288; *Homo*, AAK21848

## Supplementary Information

Below is the link to the electronic supplementary material.

Supplementary file1 (PDF 189 kb)

## Data Availability

Sequence data have been submitted to the GenBank database.
