# Supplementary material for: Complete genome sequence of a novel secovirid infecting cassava in the Americas
Source: Arch Virol. 2022 Jan 3;167(2):665–8. doi: 10.1007/s00705-021-05325-2 (PMC8844172; doi:10.1007/s00705-021-05325-2)
Supplement: Supplementary file 1 — Supplementary file1 (PDF 189 kb) [file 705_2021_5325_MOESM1_ESM.pdf]

ORF1-RNA2

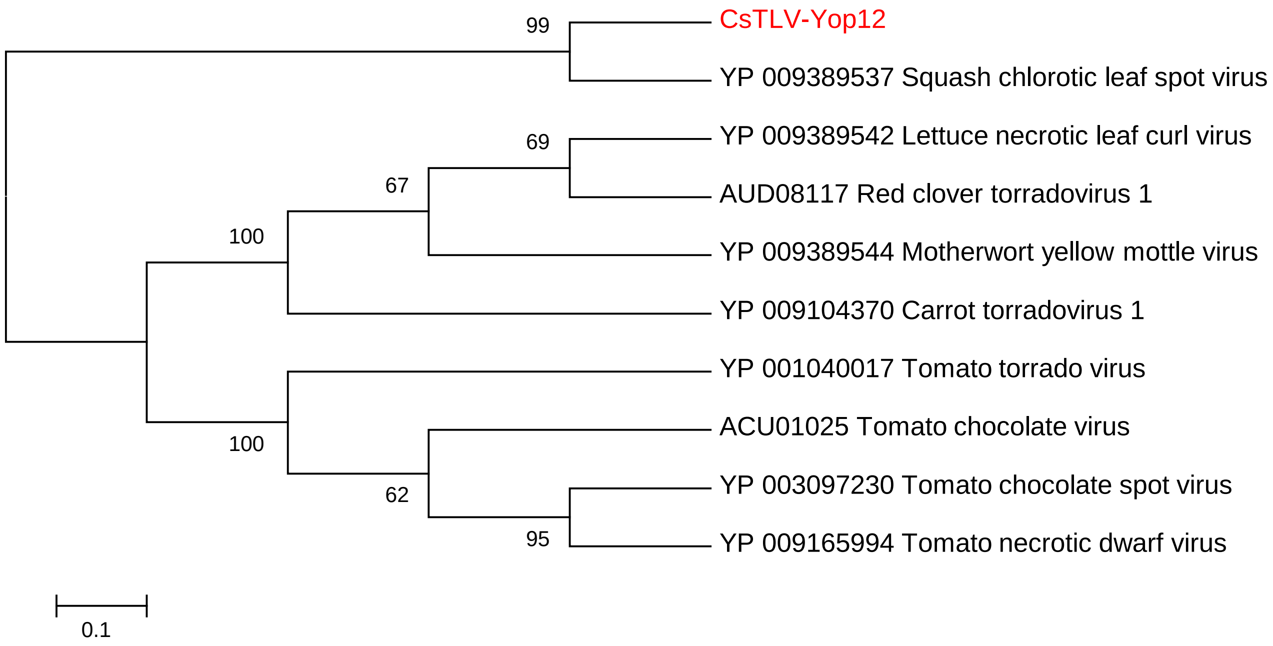

Supplementary Figure 1. Phylogenetic relationship of RNA2-ORF1 of CsTLV-Yop12 analyzed using MEGA. Phylogenetic tree generated using Neighbor-Joining method and aa sequences. The evolutionary distances were computed using the Poisson correction method and are in the units of the number of aa substitutions per site.
